# Step-Up Approach for Sodium Butyrate Treatment in Children With Congenital Chloride Diarrhea

**DOI:** 10.3389/fped.2021.810765

**Published:** 2022-01-20

**Authors:** Lavinia Di Meglio, Giusi Grimaldi, Francesco Esposito, Monica Gelzo, Maria Valeria Esposito, Giuseppe Castaldo, Roberto Berni Canani

**Affiliations:** ^1^Department of Translational Medical Science, University Federico II, Naples, Italy; ^2^Diagnostica Ecografica e Prenatale Aniello Di Meglio, Naples, Italy; ^3^Genetic Engineering and Advanced Biotechnology Center (CEINGE) Advanced Biotechnologies Research Center, University Federico II, Naples, Italy; ^4^Task Force for Microbiome Studies, University Federico II, Naples, Italy

**Keywords:** SLC26A3, butyrate, congenital diarrheal disorders, COEDS, congenital chloride diarrhea

## Abstract

**Objectives:**

Oral salt substitutive therapy is pivotal for the survival of patients with congenital chloride diarrhea (CLD), however this therapy is unable to influence the symptoms severity. Butyrate has been proposed to limit diarrhea severity in CLD. Unfortunately, the optimal dose schedule is still largely undefined. In addition, butyrate seems not to be well-tolerated by all patients, with some subjects reporting diarrhea worsening. We investigated the efficacy of a step-up therapeutic approach with sodium butyrate in patients who experienced a diarrhea worsening or an absent improvement after the direct administration of 100 mg/kg/day of sodium butyrate.

**Methods:**

The efficacy of a step-up therapeutic approach starting from 50 mg/Kg/day with a subsequent 25 mg/kg/day weekly increase up to 100 mg/kg/day of oral sodium butyrate was investigated in previously three unresponsive CLD children.

**Results:**

The step-up therapeutic approach resulted effective in limiting diarrhea severity in all our three previously unresponsive CLD patients.

**Conclusions:**

Our results suggest the efficacy of the step-up therapeutic approach in CLD children.

## Introduction

Congenital chloride diarrhea (CLD) is a rare autosomal recessive (AR) disorder caused by a mutation in SLC26A3(Solute carrier family 26, member3) gene that encodes for Down Regulated in Adenoma (DRA), an intestinal Na^+^ independent Cl^−^/${\text{HCO}}_{3}^{-}$ (or OH^−^) exchanger (1). The main feature of this condition is a severe chronic osmotic diarrhea that causes a secondary volume contraction with the development of metabolic alkalosis, electrolytes imbalance, failure to thrive, psycho-motor delay and even death (2). Oral salt substitutive therapy is a life-saving therapy: it improves the outcome, leads to electrolytes balance, reduces the risk of developing metabolic alkalosis, but has no effect on symptoms' severity (3). The quality of life in CLD patients is negatively influenced by the elevated number of evacuations a day and the fecal incontinence (4). Many symptomatic therapies have been proposed including proton pump inhibitors (PPIs), corticosteroids, cholestyramine, spironolactone (3–9). However, for many of them the effect is not well established (3–9). More recently, sodium butyrate emerged as a promising strategy to limit diarrhea severity in CLD patients (10). Sodium butyrate enhances Cl^−^ absorption through two different pathways. The first is DRA-unrelated: butyrate is absorbed by the Cl^−^/Butyrate co-transporter, stimulates the putative anion transporter1 (PAT-1), Na^+^/H^+^ exchangers 2 (NHE2) and 3 (NHE3) and inhibits the Na-K-2Cl co-transporter. The second is DRA-related. It acts on the transcription and translation of SLC26A3 by the activation of YY1 and GATA (4, 8, 11). The final result is a reduction of DRA mis-trafficking and mis-folding through cAMP inhibition (4, 8, 11). The clinical use of sodium butyrate is still limited. In some cases, diarrhea worsening has been reported in CLD patients (4, 8, 12). Genotype dependency has been hypothesized as responsible for butyrate response although also other factors could be implicated (8). Butyrate is commonly used at the dose of 100 mg/Kg/day. However, a step up therapeutic approach starting from 50 mg/Kg/day with subsequent 25 mg/kg/day weekly increase up to 100 mg/kg/day resulted also effective (4).

To address this aspect, we evaluated the efficacy of sodium butyrate administered by the step-up therapeutic approach in previous unresponsive CLD patients.

## Methods

### Ethics

The study protocol, the subject information sheet, the informed consent form, and the clinical chart were reviewed and approved by the Ethics Committee of our University of Naples Federico II (n. 3469/07) and by the Italian Medicines Agency (AIFA). The study was conducted in accordance with the Helsinki Declaration (Fortaleza revision 2013), the Good Clinical Practice Standards (CPMP/ICH/135/95), and the current Decree-Law 196/2003 regarding personal data and all the requirements set out in the European regulations on this subject.

### Study Center and Population

The Department of Translational Medical Science at the University of Naples “Federico II” is a tertiary Center for Pediatric Gastroenterology and an International Reference Center for pediatric patients with CLD. From January the 1st 2005 to January the 1st 2020, 50 cases of suspected CLD were referred to the Center, and a definitive diagnosis of CLD was obtained in 40 patients with different ethnicity. The sure diagnosis of CLD was based on the results of full anamnestic, clinical and laboratory evaluation (including the evaluation of fecal Na^+^, Cl^−^, and K^+^), and the result of the molecular analysis.

Demographic, clinical, and laboratory data of all CLD patients were collected in a dedicated database. Of the 40 CLD patients that have been followed at our center, seven patients met the inclusion criteria and were invited to participate in the study. Inclusion criteria were: a definitive diagnosis of CLD and a previous unresponsive butyrate treatment defined as an unchanged or worsted diarrhea severity (increase in the number of evacuations/day and reduction of stool consistency) during the seven days following the start of 100 mg/kg/day butyrate therapy in absence of other conditions, good compliance to salt substitution, hydro electrolyte balance with standard therapy, a wash-out period form the last butyrate administration of at least 4 weeks. Exclusion criteria were: age >18 years; severe dehydration; the concomitant presence of other acute (infectious diseases) or chronic conditions (severe allergic diseases, eosinophilic disorders of the gastrointestinal tract, congenital cardiac defects, renal insufficiency, tuberculosis, autoimmune diseases, genetic and metabolic diseases, immunodeficiencies, chronic inflammatory bowel diseases, celiac disease, lactose intolerance, obesity, neuropsychiatric and neurological disorders, cystic fibrosis, malignancy, chronic pulmonary diseases, malformations of the gastrointestinal and/or respiratory and/urinary tract); the history of gastrointestinal tract surgery; butyrate therapy in the last month, the concomitant or a <4 weeks use of probiotics, antibiotics, proton pump inhibitors, anti-diarrheal drugs; insufficient reliability or presence of conditions that made the patient's compliance with the protocol unlikely; participation into other studies.

### Molecular Analysis of SLC26A3 Gene

Molecular analysis was performed at the laboratory of CEINGE-Advanced Biotechnologies, Naples, Italy. DNA was extracted from an EDTA blood sample with the Nucleon BACC2 kit (Amersham Biosciences, USA). The primers used were reported elsewhere (13). The touchdown PCR protocol that enables co-amplification of all exons under the same PCR conditions is available on request. Sequencing analysis was carried out on both strands with an automated procedure (3100 Genetic Analyzer, Applied Biosystem). All PCR fragments were sequenced with the primers used for PCR. Furthermore, we used the Expand Long Template PCR System (Roche, Germany) to verify the deletion extension in a patient bearing c.2008-151_2061+1546 del mutation. We used the forward primer of exon 17 and the reverse primer of exon 19 (14), both known to be intact in exon-specific assays. The expected fragment is about 6300 bp. The PCR conditions are available on request.

### Study Design

**Figure 1:**
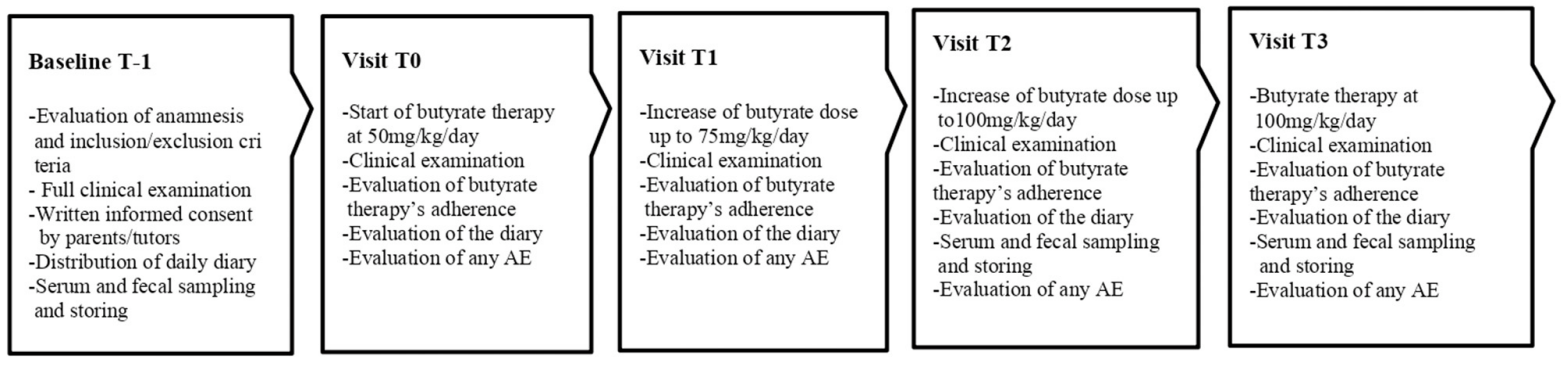
The design of the step-up therapeutic approach study.

The step up therapeutic approach was proposed in CLD subjects unresponsive to butyrate treatment, defined as patients who experienced an unchanged or worsted diarrhea severity (increase in the number of evacuations/day and reduction of stool consistence) during seven days following the start of 100 mg/kg/day butyrate therapy. The Ethics Committee of our University approved the study protocol. The study design is depicted in Figure 1.

In these patients we used a commercially available sodium butyrate formulation. The starting dose was 50 mg/Kg/day divided in two administrations, with subsequent weekly 25 mg/kg/day increase up to the final dose of 100 mg/kg/day divided in two administrations (4).

The primary outcome was the reduction in the daily number of evacuations.

The secondary outcomes were the improvement of stool consistency evaluated by the Bristol Stool Chart (BSC) and the reduction of fecal Na^+^, Cl^−^ lose (4, 15).

The purposes and the modalities of the study were illustrated to the patients and their parents/tutors during the first visit (T-1), and written informed consent was obtained from parents or tutors of each enrolled patient. At baseline (T-1), a full clinical evaluation (including body weight and height, blood pressure, pulse rate and electrocardiogram) was performed and blood, urine and fecal samples were collected for the determination of the following parameters: blood cell counts, serum creatinine, serum renin and aldosterone; urine analysis; and fecal Na^+^ and Cl^−^. History of any other previous additional therapies were collected. A daily diary was hand over to the parents where they had to report the daily number of evacuations and the stool consistency score from that day on, the diary was analyzed during each visit and was recollected at T3. Parents were instructed on how to report and evaluate BSC. From T-1 to T0 the baseline number of evacuations a day was calculated. After 7 days (T0) butyrate therapy with step up approach was started, and a weekly evaluation of stool pattern and adherence to butyrate treatment was planned. When the final dose of 100 mg/Kg/day was achieved (T2) of butyrate and then, for the following 6 months (T3) the clinical features and adherence data of the patients were re-evaluated, and blood, urinary and fecal values were monitored.

The parents were informed that in case of any adverse events, concomitant pathologies, or therapies they had to contact the center immediately.

### Data Extraction and Risk of Bias Assessment

Two authors (LDM, RR) reviewed all clinical records independently and extracted the data independently. In case of different results, a third author (GG) was consulted.

### Data Collection and Analysis

The Kolmogorov-Smirnov test was used to determine whether variables were normally distributed. Descriptive statistics were reported as means and standard deviations for continuous variables or median and interquartile range (IQR), and discrete variables were reported as the number and proportion of subjects with the characteristic of interest. The χ2 test and Fisher's exact test were used for categorical variables. To evaluate the differences among continuous variables, the independent sample *t*-test were performed. The level of significance for all statistical tests were 2-sided, *p* < 0.05. All data were collected in a dedicated database and analyzed by a statistician blinded to patient group assignment, using SPSS for Windows (SPSS Inc, version 14.0, Chicago, IL).

## Results

### Efficacy of Step-Up Therapeutic Approach

A total of 7 patients were evaluated for the study based on the including criteria, in two subjects the possibility of participating in the study was impeded by logistical reasons (residence in another country), two were excluded for the presence of at least one exclusion criteria (poor compliance to basal therapy, concomitant chronic condition). A total of three subjects were enrolled. Main demographic and clinical characteristics are resumed in Table 1. All the patients were taking salt substitution according to their weight and were well-controlled by basal therapy (3). PPI was previously administered in patient #001 at the age of 28 months for eight consecutive weeks without improvement.

**Table 1:** Baseline features of previous unresponsive to butyrate therapy CLD patients.

**Patient's code**	**#001**	**#002**	**#003**
Gender	M	M	F
Age at diagnosis, months	26 m	1 m	8 m
Age at enrollment, years	32 m	9 y and 4 m	7 y and 5 m
Ethnicity	Caucasian	Caucasian	Caucasian
Body weight, kg (centile)	15.0 (50°)	20.9 (<3°)	25.0 (90°)
SLC26A3 mutation	Nonsense	Nonsense	Splicing
Age at first butyrate therapy attempt (effect)	2 y and 2 m (Unchanged diarrhea severity)	2 y (Worsted diarrhea severity)	7 y (Unchanged diarrhea severity)

*M, male; F, female; y, years; m, months*.

**Figure 2:**
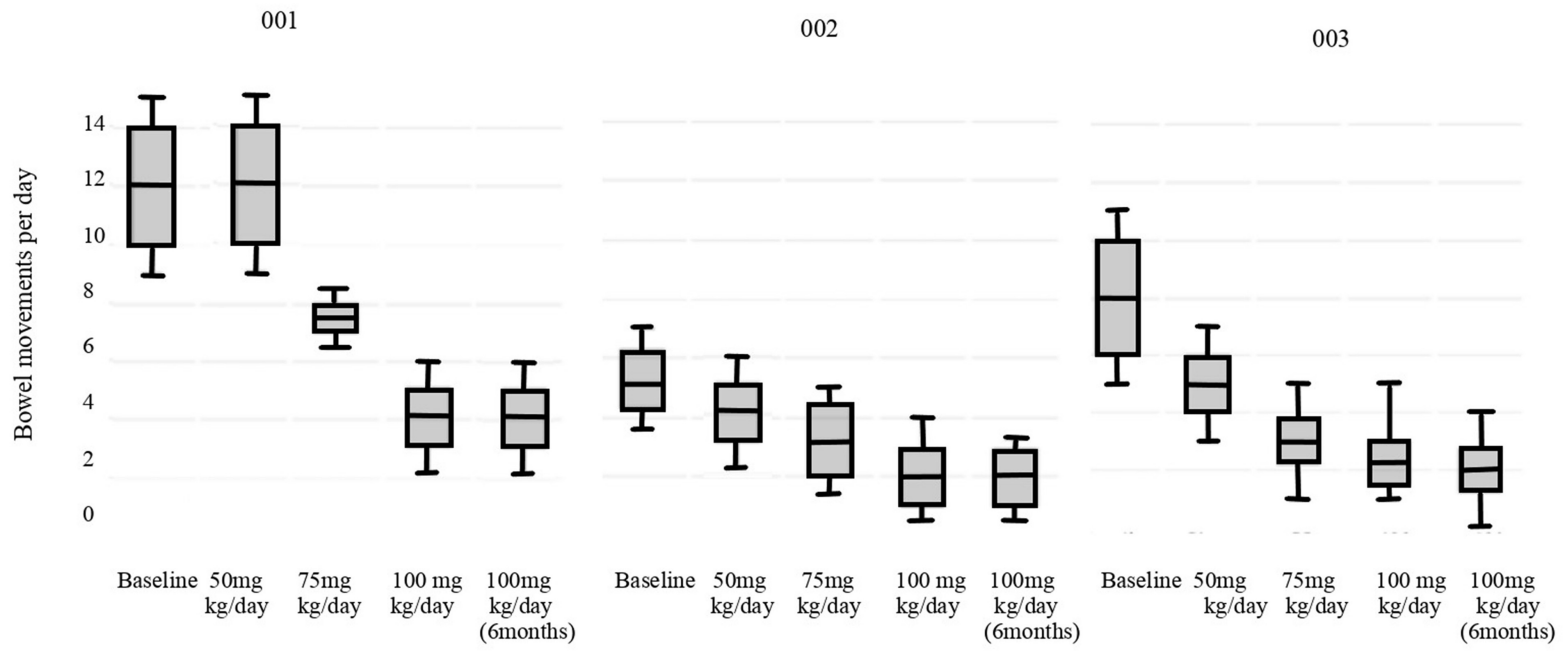
Primary outcome results.

The results of primary outcomes are presented in Figure 2 (*p* < 0.05). All the patients reached the physiological daily number of evacuations. Stool consistency score at baseline (T-1) was evaluated as seven according to the BSC for all patients. For patients #002 and #003 at 100 mg/Kg/day of sodium butyrate and after 6 months the consistence was 6, for patient #001 it was unchanged. Fecal sodium baseline values were 74 mEq/L for patient #001, 71 mEq/L for #002 and 70 mEq/L for #003; at 100 mg/kg/day of butyrate were respectively 65 mEq/L, 50 mEq/L, 75 mEq/L (*p* = 0.19). Chloride baseline values were 149 mEq/L for patient #001, 150 mEq/L for #002 and 169 mEq/L for #003, at 100 mg/kg/day of butyrate were respectively 103 mEq/L, 102 mEq/L, 100 mEq/L (*p* = 0.008). No adverse events were reported in any of the cases.

## Discussion

By a literature review performed on PUBMED and EMBASE, the effect of butyrate therapy has been reported in 18 CLD patients so far (4, 8, 10, 12, 14, 16–19) (Table 2). Just one patient was successfully treated by a step-up therapeutic approach, whereas to the other 17 patients butyrate therapy was administered starting with 100 mg/kg/day and they presented conflicting responses, and a possible genotype dependent response has been suggested (4, 8). For this reason, we planned to evaluate the therapeutic efficacy of butyrate using the step-up approach in previous non-responder patients. We hypothesized that the gradual increase in butyrate daily dose could be able to stimulate a more stable modulation of PAT-1, NHE2-3 and DRA activity with a parallel stronger tolerably of its use (4, 8, 11, 20–22). Sodium butyrate could enhance Cl^−^ absorption by the DRA related pathway in a more strong way acting directly on the transcription and translation. In all our previous non-responsive patients, the step-up therapeutic approach was well-tolerated and resulted in a reduction of bowel movements and fecal Cl^−^ losses. These results suggest that more unknown mechanisms, besides genotype-efficacy relationship, could be involved in butyrate therapeutic efficacy in CLD patients (4, 8, 10, 12).

**Table 2:** Main data reported in literature on butyrate (100 mg/Kg/day) use in CLD.

**Authors, case number**	**Age (year)**	**Genotype**	**CLD complication**	**Response**	**AE**
Fuwa (16), #004	4/12	c.2063-1G>T Splicing c.392C>T Splicing	Diarrhea	Unchanged diarrhea severity	None
Berni Canani (8), #005	16	c.1484A > C Missense c.1640C >A Missense	Diarrhea	Improvement of stool pattern with reduced bowel movements number, stool volume and fecal Cl^−^ loss.	None
Berni Canani (8), #006	3	c.386C > T Missense	Diarrhea	Improvement of stool pattern with reduced bowel movements number, stool volume and fecal Cl- loss.	None
Berni Canani (8), #007	12	c.1008-151_2061 + 1546del Deletion	Diarrhea	Improvement of stool pattern with reduced bowel movements number, stool volume and fecal Cl- loss.	None
Berni Canani (8), #008	18	c.2132 T>G Non-sense	Diarrhea	Unchanged diarrhea severity	None
Berni Canani (8), #009	18	c.559G >T Non-sense	Diarrhea	Unchanged diarrhea severity	None
Berni Canani (8), #010	15	c.559G >T Non-sense	Diarrhea	Unchanged diarrhea severity	None
Berni Canani (8), #011	15	c.1408-G >C Splicing	Diarrhea	Unchanged diarrhea severity	None
Berni Canani (4), #012	11	c.Q495H missense c.A547E missense	Diarrhea, multiple hospital admissions	Improvement of stool pattern with reduced bowel movements number, stool volume and fecal Cl^−^ loss.	None
Egritaş (14), #013	1 and 8/12	c.559G>t non-sense	Diarrhea, failure to thrive	Unchanged diarrhea severity	None
Wedenoja (12), #014	8	c.V317del Deletion	Diarrhea	Improvement of stool pattern with reduced bowel movements number, stool volume and fecal Cl- loss.	None
Wedenoja (12), #015	12	c.V317 Deletion	Diarrhea	Unchanged diarrhea severity	None
Wedenoja (12), #016	1 and 8/12	c.V317 Deletion	Diarrhea	Diarrhea worsening	None
Wedenoja (12), #017	1 and 9/12	c.V317 Deletion	Diarrhea	Diarrhea worsening	None
Wedenoja (12), #018	1 and 11/12	c.V317 Deletion	Diarrhea	Diarrhea worsening	None
Gujrati (17), #019	1 and 4/12	Not available	Diarrhea, failure to thrive	Improvement of stool pattern with reduced bowel movements number, stool volume and fecal Cl^−^ loss, body weight gain	None
Mushtaq (18), #020	5/12	Not available	Diarrhea	Decreased bowel movements number	None
Sajid (19), #021	7/12	Not available	Diarrhea, multiple admissions, failure to thrive	Improvement of stool pattern with reduced bowel movements number, stool volume and fecal Cl^−^ loss, body weight gain.	None

*AE, Adverse events*.

In addition, the step-up therapeutic approach allows a daily monitoring of butyrate response and the identification of the minimum effective dose. For example, patient #002 reached the physiological number of evacuation a day (<3 bowel movements daily) at 75 mg/kg/day (23).

By now, the length of follow up is up to 2 years for patients #002 and #003 and of 18 months for patient #001, they still present <3 normal evacuations daily and, by now, they have never experienced butyrate intolerance.

The main limitation is the small number of patients that participated to our study due to the strict inclusion criteria and the rarity of this disease; however, this study represents a starting point to subsequent multicenter and larger studies.

## Conclusion

The butyrate step-up therapeutic approach could be an effective strategy for all CLD patients independently from the genotype. This approach could facilitate the monitoring of the patient week by week until the optimal dose is reached. A first unsuccessful butyrate's therapeutic course could not be a contraindication for this approach. Butyrate therapy could lead to long term and safe control of diarrhea severity in CLD patients.

## Data Availability Statement

The original contributions presented in the study are included in the article/supplementary material, further inquiries can be directed to the corresponding author.

## Ethics Statement

The studies involving human participants were reviewed and approved by Ethics Committee University of Naples Federico II. Written informed consent to participate in this study was provided by the participants' legal guardian/next of kin.

## Author Contributions

LD, GG, FE, and RB wrote the original draft and contributed to conception and design of the manuscript. MG, ME, and GC performed data acquisition, analysis, and interpretation. All authors reviewed critically the manuscript, edited the final draft, approved the final version of the article, and including the authorship list.

## Funding

This research was funded by a research grant provided by the Italian Medicines Agency (AIFA project–code MRAR08W002).

## Conflict of Interest

The authors declare that the research was conducted in the absence of any commercial or financial relationships that could be construed as a potential conflict of interest.

## Publisher's Note

All claims expressed in this article are solely those of the authors and do not necessarily represent those of their affiliated organizations, or those of the publisher, the editors and the reviewers. Any product that may be evaluated in this article, or claim that may be made by its manufacturer, is not guaranteed or endorsed by the publisher.
